# HGTree v2.0: a comprehensive database update for horizontal gene transfer (HGT) events detected by the tree-reconciliation method

**DOI:** 10.1093/nar/gkac929

**Published:** 2022-11-09

**Authors:** Youngseok Choi, Sojin Ahn, Myeongkyu Park, SaetByeol Lee, Seoae Cho, Heebal Kim

**Affiliations:** Department of Agricultural Biotechnology and Research Institute of Agriculture and Life Sciences, Seoul National University, Seoul 08826, Republic of Korea; Interdisciplinary Program in Bioinformatics, Seoul National University, Seoul 08826, Republic of Korea; eGnome Inc., Seoul 05836, Republic of Korea; Interdisciplinary Program in Bioinformatics, Seoul National University, Seoul 08826, Republic of Korea; eGnome Inc., Seoul 05836, Republic of Korea; eGnome Inc., Seoul 05836, Republic of Korea; Department of Agricultural Biotechnology and Research Institute of Agriculture and Life Sciences, Seoul National University, Seoul 08826, Republic of Korea; Interdisciplinary Program in Bioinformatics, Seoul National University, Seoul 08826, Republic of Korea; eGnome Inc., Seoul 05836, Republic of Korea

## Abstract

HGTree is a database that provides horizontal gene transfer (HGT) event information on 2472 prokaryote genomes using the tree-reconciliation method. HGTree was constructed in 2015, and a large number of prokaryotic genomes have been additionally published since then. To cope with the rapid rise of prokaryotic genome data, we present HGTree v2.0 (http://hgtree2.snu.ac.kr), a newly updated version of our HGT database with much more extensive data, including a total of 20 536 completely sequenced non-redundant prokaryotic genomes, and more reliable HGT information results curated with various steps. As a result, HGTree v2.0 has a set of expanded data results of 6 361 199 putative horizontally transferred genes integrated with additional functional information such as the KEGG pathway, virulence factors and antimicrobial resistance. Furthermore, various visualization tools in the HGTree v2.0 database website provide intuitive biological insights, allowing the users to investigate their genomes of interest.

## INTRODUCTION

Genetic materials are often inherited from parents to their offspring vertically, but for prokaryotes such as bacteria or archaea (that reproduce asexually), the inheritance can be demonstrated horizontally during species interactions, from one adjacent organism to another, for their evolutionary benefit (1). The asexual reproduction of prokaryotes gives nearly identical genetic information to the offspring, making horizontal gene transfer (HGT) an essential process in achieving genetic variations (2). HGT is one of the driving forces responsible for shaping the prokaryotic genomes and surviving natural selection (3). For example, taking up genes responsible for antimicrobial resistance could be critical in facilitating the organism's adaptation to a particular environment. Contrarily, organisms that have taken up unnecessary genes have more chances of excluding themselves from natural selection if the genes are either neutral or detrimental (4). As much as HGT contributes to the evolution of prokaryotic organisms, it can always complicate the interpretation of the lineage or the history of species evolution, leading to an erroneous result regarding their classifications. Thus, it is fundamental in the research of prokaryotic evolution to understand HGT in depth (5).

In view of this, we constructed HGTree (https://hgtree.snu.ac.kr) (6) in 2015. Other conventional databases such as the HGT-DB (7) or DarkHorse HGT Candidate Resource (8) utilize genome signatures (such as GC bias, nucleotide composition and codon usage) or implicit phylogenetic methods (such as comparison of evolutionary distance derived by sequence similarity). Unlike them, HGTree exploits the tree-reconciliation method, an explicit phylogenetic method that uses species trees and corresponding gene trees to determine horizontally transferred genes. The tree-reconciliation method is well known for its reliability and is a prevailing method for HGT event detection (9,10). and the HGTree database was able to implement the method while overcoming its challenges (6). As a result, the database was built with 660 840 HGT event results of 2472 prokaryotic genomes.

Since 2015, the amount of prokaryotic genome data has increased dramatically, and so has the requirement for a more comprehensive HGT database with more reliable and efficient processes to detect HGT events. After the publication of HGTree, new methods to detect HGT events have been continuously developed. ShadowCaster hybridized the implicit method with the support-vector-machine (SVM) method (11), and tools such as NearHGT (12) or RecentHGT (13) were developed to detect HGT events between closely related taxa. However, these tools are not suitable for accurately detecting the HGT events among various phyla, leaving the tree-reconciliation method as the most reliable detection method as long as accurate inference trees are supported (14).

Therefore, we present a newly updated HGTree database in response to these demands, coping with the growing interest in HGT. The updated HGTree (referred to as HGTree v2.0) includes approximately eight times larger genomes than the previous version, yielding approximately over six times more putative HGT event results. The newly revised HGT events detection procedure ensured increased detection reliability, and the detected events are presented in the HGTree v2.0 website equipped with more user-friendly interfaces, including the modified user query processor, which allows users to navigate their genomes.

## DATA COLLECTION AND PROCESSING

### Data processing

The genome data used in this research were retrieved from NCBI using two different search titles: ‘Bacteria’ and ‘Archaea’. Further options included ‘Assembly’, ‘Latest RefSeq’ (15) and ‘Complete genome’ (https://www.ncbi.nlm.nih.gov/assembly; May 5, 2021) (16). A total of 20 179 bacterial and 357 archaeal genomes were retrieved at the strain level. Along with the nucleotide and amino acid sequence data, GenBank data were also retrieved, including each genome's size, gene function and the number of coding sequences (CDSs). The taxonomy of each genome was further confirmed and retrieved from the GTDB database (https://gtdb.ecogenomic.org/) (17).

To detect orthologous genes, 20 179 bacterial genomes and 357 archaeal genomes were processed separately using PorthoMCL (18). All-versus-all blast search was performed to detect orthologous genes, and the genes were clustered and assigned to orthology groups. We set the alignment coverage to 80%, an E-value cut-off to 10^–6^ and the minimum identity score was 98%. To retrieve 16S rRNA, RNAmmer (ver. 1.2) (19) was used. The program uses an HMMER- (ver. 3.1b2) (20) based scanning procedure to detect 16S rRNA of both bacteria and archaea from the input genome data file.

For each detected orthology group and its corresponding 16S rRNA group, CLUSTAL Omega (ver. 1.2.3) (21) was used for the sequence alignment, and FastTree2 (ver. 2.1.9) (22) was used to construct phylogenetic gene and species trees. As a result, an unrooted gene tree (made of orthologous protein sequences) and a species tree (made of 16S rRNA sequences of genomes from the same orthology group) were constructed for each orthology group. Newick Utility (newick_utils ver. 1.6) was used to re-root the species trees using the 18S rRNA sequence from *Saccharomyces cerevisiae* for the outgroup.

Ranger-DTL 2.0 (23) was used to detect horizontally transferred genes among different orthologous group sets of phylogenic trees. The first round of Ranger-DTL 2.0 was done under default parameters (duplication cost, 2; transfer cost, 3; loss cost, 1) for a standard result. Next, we ran a second round with ‘3’ and ‘4’ for the transfer cost for a more rigorous analysis, leaving the duplication and loss cost as default. The two different transfer cost results were then aggregated as one result in the AggregateRanger step. The results of the first and second rounds were named as the ‘Standard DB’ and the ‘Strict DB’, respectively. This process will be further elucidated in the Results and Discussion.

The 20 536 genomes were scanned against PfamScan (ver. 1.6) (24) under the default parameter to conduct protein family-level assignment. DIAMOND (ver. 0.9.14) (25) BLASTP (ver. 2.2.31+) (26) search was conducted with all detected putative HGT-related genes against the VFDB database (http://www.mgc.ac.cn/VFs/main.htm) (27) to classify virulence factors, and CARD-rgi (ver. 5.2.1) (28) was conducted to identify antimicrobial resistance genes. For the annotation of the KEGG (Kyoto Encyclopedia of Genes and Genomes) pathway, the emapper command from the software eggNOG (eggNOG-mapper ver. 2.1.7) (29,30) was used. The annotation of COG clusters was done by running BLASTP against the COG database (31) .

### User query processor

**Figure 1.**
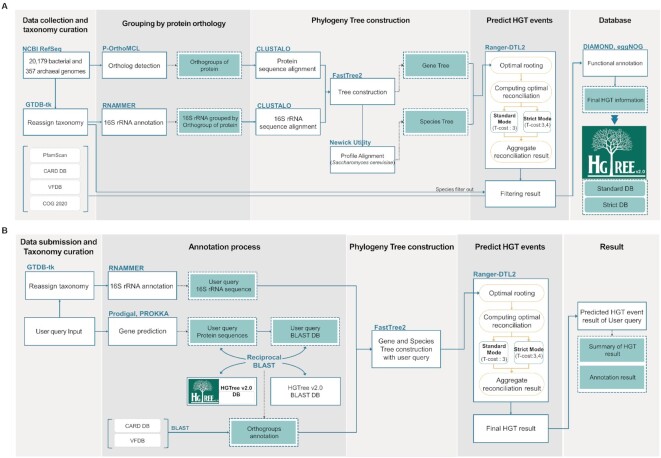
The flowchart of constructing the HGTree v2.0 database and user query process. (**A**) The overall workflow of constructing the HGTree v2.0 database. To construct the database, 20 179 bacterial and 357 archaeal complete genomes were downloaded from the NCBI. The taxonomy of each genome was reclassified using GTDB-tk. The orthology groups were calculated with POrthoMCL and each group was aligned for phylogenetic gene tree construction. Corresponding species trees were made with 16S rRNA sequences of genomes in the orthology group. Each group's gene tree and species tree are used to perform RANGER-DTL, and putative HGT events and related genes are detected. HGT-related genes’ Pfam, antimicrobial resistance, virulence factors, COG clusters and KEGG pathway annotations are also uploaded to the database. (**B**) The overall algorithm of the user query processor. Users can upload their prokaryotic complete genomes to analyze HGT-related genes in them. In the user query processor, reciprocal blast exploits the HGTree v2.0 database results to calculate new orthology groups that include genes of the input genome. Other parts of the workflow follow the procedures of the previous version, except that users can choose the processor mode (the ‘Standard Mode’ and ‘Strict Mode’).

We also modified the user query processor of the previous version, and the steps are as follows: first, Prokka (ver. 1.11) (32) and Barrnap (ver. 0.9) (33) are used to predict protein sequences and 16S rRNA of the submitted genome. Second, both the protein sequences of the query genome and HGTree v2.0 data are used to generate two BLAST databases to find the best-hit results by performing the reciprocal BLAST search against each other. For the HGTree v2.0 BLAST database to be used, users can choose between the ‘Standard DB’ and ‘Strict DB’ for the BLAST search. The default parameters for the reciprocal BLAST are set as 80% for alignment coverage, 80% for identity score and an E-value cut-off of 10^–6^, but users can choose their parameters too. The best-hit results will be assigned to HGTree v2.0 database orthology groups to generate new orthology groups that include sequences of the query genome. Third, gene trees and corresponding species trees are generated through ClustalO and FastTree2. Finally, Ranger-DTL2 is performed to detect putative horizontally transferred genes from the input genome. In the same way as we built the HGTree v2.0 database, if users choose ‘Strict Mode’ in the second step, Ranger-DTL will run twice, once with default transfer cost and the secondly time with transfer cost of ‘3’ and ‘4’. Users will be given a final text file stating the ratio of HGT-related gene events and a list of them, separated into two sections: ‘Donated Genes’ and ‘Received Genes’. Virulence factors, antimicrobial resistance, gene names, product names and the genomes of the donors and recipients will also be included in the text file. The workflows of both database construction and the user query processor are illustrated in Figure 1.

### Website visualization

The MariaDB (ver. 10.5.8) (http://mariadb.org/) management system was used. The web-based user interface was generated with HTML5, django, CSS and JavaScript. DataTable (ver. 1.11.5) (https://datatables.net/) and jQuery (ver. 2.1.1) (http://jquery.com) were used to implement the user interface widgets. To generate circular phylogenetic trees, jsPhyloSVG-1.55 (34) was used and graphics were illustrated with Google Chart SVG, JavaScript library, D3 (35).

## RESULTS AND DISCUSSION

**Figure 2.**
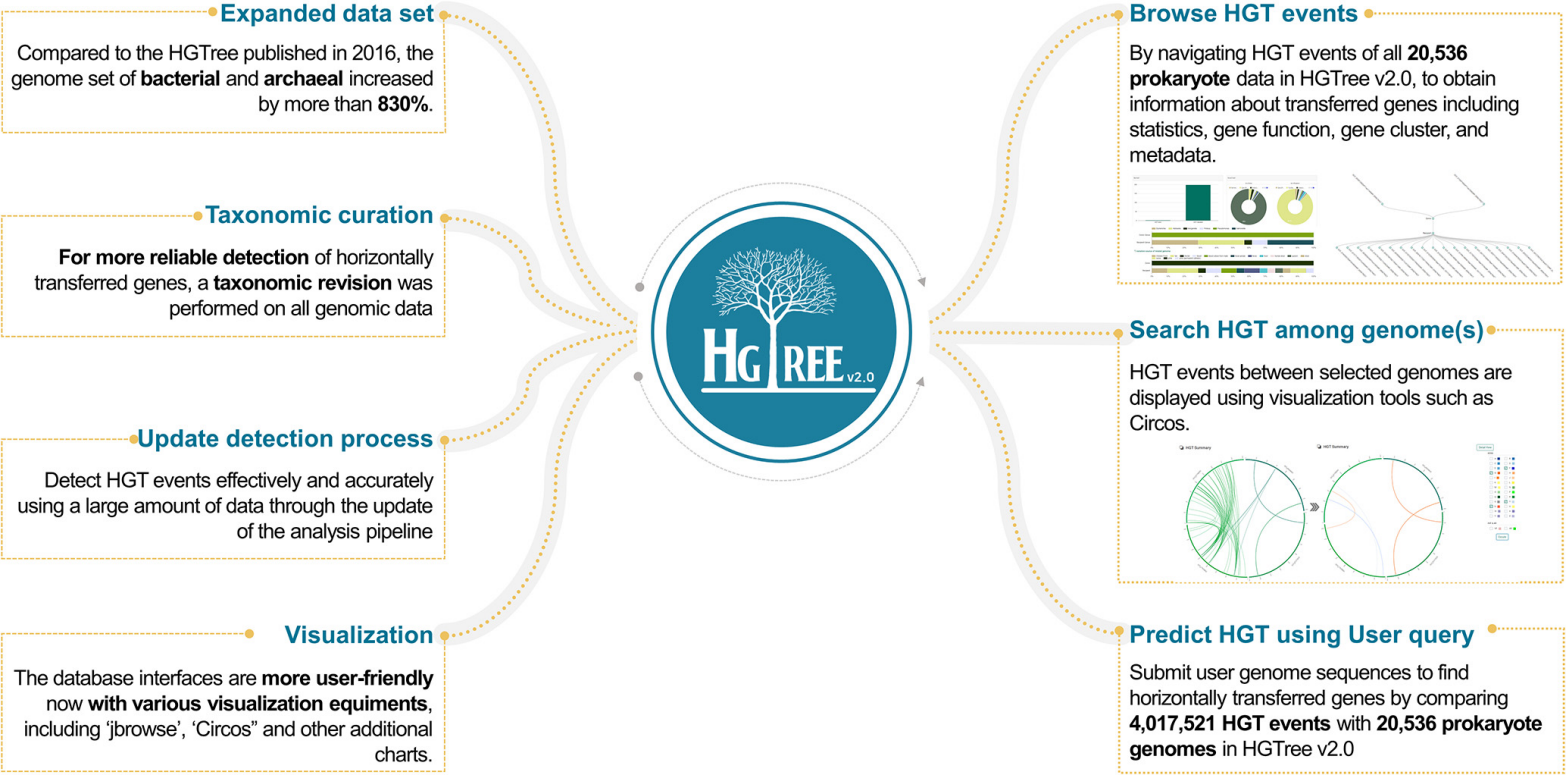
The overview of HGTree v2.0. HGTree v2.0 has expanded datasets, changed the pipeline of HGT event detection, added more curations steps in detecting horizontally transferred genes and is equipped with more visualization equipment for users. Users can ‘Browse’ and ‘Search’ HGT events and their related genes among different types of genomes in our vast database and can upload their genomes to find out what genes are engaged in HGT events.

In HGTree v2.0, to keep pace with the ever-inceasing amount of new prokaryotic genome data, we acquired information on putative HGT-related genes through a more accurate tree-reconciliation method. Our major focus of the update was to ensure that the HGT results are more reliable and to show their various functional data in more user-friendly ways. The overview of updates on HGTree v2.0 can be seen in Figure 2.

### Enhanced HGT events detection procedures

The orthology grouping step of the previous version was done using Mestortho. Mestortho is a powerful tool that uses the distance method to calculate orthology based on the phylogenetic criterion of minimum evolution (36). However, considering the vast amount of data input for constructing the database, Mestortho was replaced with PorthoMCL due to Mestortho's limitation on speed and time taken to calculate all minimum evolution of 20 536 genomes. PorthoMCL is designed to calculate orthology groups of a considerable number of genomes with its parallelized sparse file structure using a Markov Cluster algorithm (18), so we expected it to be more suitable for our dataset. PorthoMCL utilizes a similar algorithm to OrthoMCL (37) but, according to Tabari *et al.*, PorthoMCL was able to process at least a five times larger size of the input genome than OrthoMCL, while the percentage of the process speedup was up to 455%.

As the detection of horizontally transferred genes relies on the reconciliation of gene and species phylogenetic trees, clarifying each genome's exact lineage and species information is crucial. Therefore, we used a new approach by employing GTDB-tk to reclassify the taxonomy of all the genomes for more reliable detection of HGT-related genes. Furthermore, our method was not adequate for detecting HGT events between the strains of the same species since we used 16S rRNA sequences to build phylogenetic trees. Using 16S rRNA made the species trees less clear as it gets down to the strain level because 16S rRNA is a universal species marker for prokaryotes, possibly inducing false-positive results regarding HGT events between the strains from the same species. Constructing phylogenetic trees based on the whole-genome ANI method could have been an alternative, but the ANI method was considered inadequate to be used due to a decrease in discriminatory power among taxa higher than the family level (38). Taking these into consideration, the GTDB-tk reclassification of each genome was important and ensured that no such false-positive events were included in the output result. GTDB-tk was also used in the user query processor so the taxonomy information of the query genome could be reclassified.

One of the limitations the previous version had was that it missed other possibly calculable optimal reconciliations during the Ranger-DTL step. This problem was solved in the current update as Ranger-DTL 2.0 was used. In the OptRoot step, 3 028 789 pairs of trees (rooted species and unrooted gene trees), constructed by aligning the same number of orthologous gene groups, were used to calculate all optimal gene tree rootings to additionally produce 1 500 857 trees, which results in 4 529 646 pairs of trees altogether. The first version of Ranger-DTL (39) did not allow this type of optimal calculation, so the users of HGTree had to investigate their genomes with only one optimal gene tree per orthology group. The subsequent Ranger-DTL step then calculated all optimal tree-reconciliations for the gene and species trees and returned one reconciliation result that was calculated to have the minimum reconciliation cost. For the second round of the Ranger-DTL step (as mentioned above in the ‘Data collection and processing’ section), as prior research exemplified, the change in transfer costs from ‘3’ to ‘4’ resulted in the detection of more reliable HGT-related genes, but much fewer in number. Those genes also had higher risks of false negatives (40,41). However, by comparing transfer cost ‘4’ results with cost ‘3’ results during the aggregation step, the chances of false negatives could be reduced (41). This was because the AggregateRanger step aggregated all the Ranger-DTL results (per orthology group) into one result and provided the percentage of genes in each orthology group that showed 100% consistent ‘events’ and ‘mapping’ among all optimally calculated Ranger results (23). This percentage was additionally used as our filtering value and, out of all aggregated reconciliation results, those that were 90% consistent were filtered.

### Expanded database size

**Figure 3.**
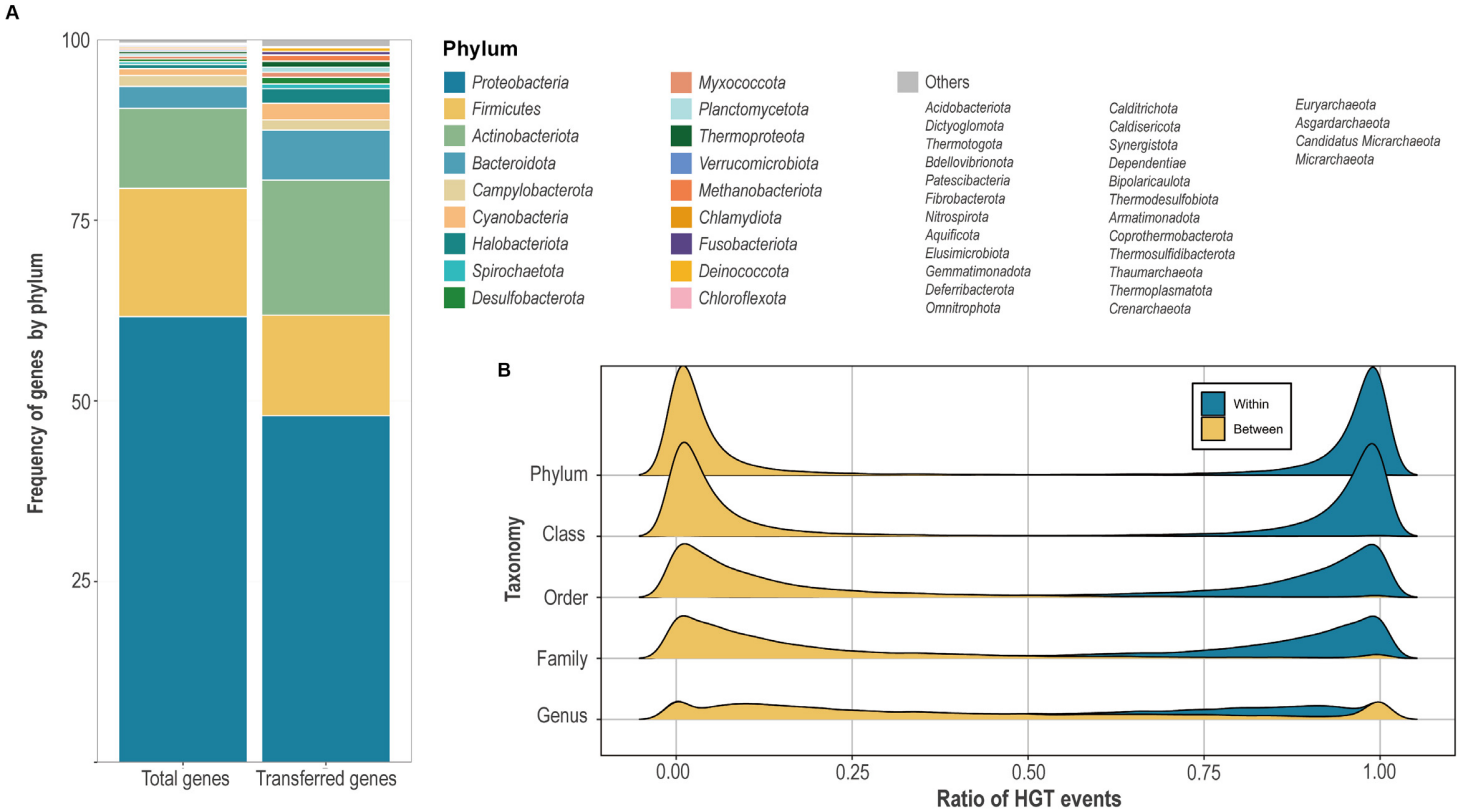
The ratio of total genes and HGT events per phylum and the ratio of HGT events which occurred between and within taxa. (**A**) The relative frequency of prokaryotic genomes’ total genes and HGT-related genes was visualized at the phylum level. More HGT-related genes were detected as the total number of genes in the phylum was larger. (**B**) The *x*-axis shows the ratio of the HGT-related genes and the *y*-axis shows the taxonomy of the animal kingdom, from the genus level to the phylum level. The yellow peaks show the ratio of HGT events between different taxa, and the blue peaks show the ratio of those that occurred within the same taxonomy. Both peaks were denser as the taxonomy levels were higher, meaning more genes were transferred between closely related organisms.

We used 20 536 non-redundant prokaryotic genomes (20 179 bacterial genomes and 357 archaeal genomes, 4964 and 264 species, respectively) to detect 3 028 789 orthologous groups and, by using them, we predicted 6 361 199 HGT-related genes (6 174 528 for bacterial genomes and 186 671 for archaeal genomes) (Table 1). As shown in Figure 3A, we could identify how many HGT-related genes were predicted per phylum, and it was clear that there was a positive correlation between the ratio of phyla and the ratio of predicted HGT-related genes. Nevertheless, some phyla such as *Actinobacteriota* and *Bacteriodota* had a higher ratio of predicted HGT events relative to their total gene ratios. Based on this result, we also calculated the frequency of HGT events among each taxon, whether the events occurred within or between taxa (Figure 3B). As the figure shows, more genes were transferred within taxa, which also concurs with previous research that HGT occurs between closely related organisms more frequently (42–44).

**Table 1.** Summary of the comparison between HGTree v2.0 and the previous version

Database		HGTree	HGTree v2.0
Total non-redundant microbial genome		2472	20 536
Genome retrieval day		17 March, 2015	5 May, 2021
No. of phyla and genera		41 and 700	36 and 1542
Total protein sequence		7 748 306	74 339 979
Number of orthologous gene sets		154 805	3 028 789
Detected putative HGT events		660 840	Standard mode: 4 017 521, Strict mode: 1 314 170
Detected putative HGT-related genes		1 401 252	Standard mode: 6 361 199, Strict mode: 1 987 538
KEGG-annotated genes		NA	1 827 784
Transferred virulence factors (for bacterial genomes)		NA	42 793
Transferred antimicrobial genes (for bacterial genomes)		NA	4544
**Programs used**			
Species re-classification		NA	GTDB-tk* (ver. 1.7.0)
Orthologous group detection		Mestortho	PorthoMCL
Sequence alignment		CLUSTAL Omega (ver. 1.2.1)	CLUSTAL Omega (ver. 1.2.3)
Phylogenetic tree construction		FastTree (ver. 2.0)	FastTree (ver. 2.1.9)
Tree reconciliation		RANGER-DTL-U (ver. 1.0)	Ranger-DTL 2.0*
**Functional annotation**	Pfam	PfamScan (ver. 1.2)	PfamScan (ver. 1.6)
	COG	COG 2014 Database	COG 2020 Database
	KEGG	NA	eggNOG-mapper (ver. 2.1.7)
	Virulence genes	NA	VFDB 2019
	Antimicrobial genes	NA	CARD-rgi (ver. 5.2.1)

* The important changes in HGTree v2.0. By employing the GTDB-tk program, all genomes' taxonomy was reclassified for more reliable results. Also, the Ranger-DTL program allowed more reliable detection of HGT events by performing all possible optimal calculations.

**Figure 4.**
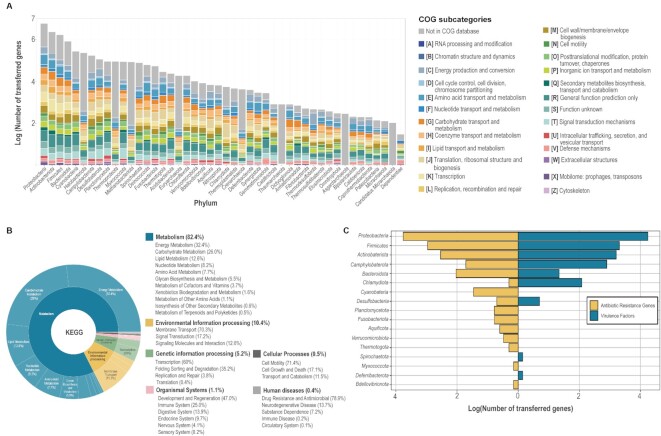
The summary of functional annotations for horizontally transferred genes. (**A**) The ratio of COG clusters for each phylum. Most of the clusters were responsible for the metabolism of the organism. A moderate portion of each genome was unclassified since no data were in the COG database. (**B**) The ratio of KEGG pathway and subcategories of all bacterial genomes used. Most of the genes were classified in the ‘Metabolism’ category, followed by ‘Environmental Information Processing’, ‘Genetic Information Processing’, ‘Organismal Systems’, ‘Cellular Process’ and ‘Human Diseases’. (**C**) The number of virulence factors and antimicrobial resistance genes of bacterial genomes.

In the previous version, only available Pfam IDs and COG (Clusters of Orthologous Genes) clusters were annotated to putative horizontally transferred genes. For a further analysis of bacterial horizontally transferred genes, the current update also focused on other functional annotations such as the KEGG (45) pathway, virulence factors and antimicrobial resistance genes. As a result, 21 961 virulence factors and 7140 antimicrobial resistance genes were identified from the HGT-related genes (Figure 4C), and 1 827 784 HGT-related genes were annotated with KEGG pathways. The frequency of each cluster is illustrated in Figure 4A and B. Interestingly, a considerable number of genes were responsible for the ‘Metabolism’ of the organism, followed by ‘Environmental Information Processing’, ‘Genetic Information Processing’, ‘Organismal Systems’, ‘Cellular Process’ and ‘Human Diseases’. Most genes responsible for ‘Metabolism’ were related to ‘Energy Metabolism’ and ‘Carbohydrate Metabolism’.

In addition, the metadata of each genome were also retrieved from the NCBI database, which includes the ‘Country’, ‘Isolation Source’ and ‘Collection Date’. The metadata are displayed on the website together with the world map (further explained in the following ‘Upgraded data visualization’ section) and will provide further geographical insights to the users.

### Upgraded data visualization

In the current update, we revised the website interface so that users can interpret results more thoroughly and quickly. The ‘Browse’ section provides all putative HGT events and the genes of 20 536 genomes. It also displays other information such as the genomes’ taxonomy, isolation source, geographical location and the ratio of HGT-related genes in the genome (the HGT index). Users can choose a genome in the database to see its HGT-related genes (genes in ‘Standard DB’ and ‘Strict DB’, separately) and can also view charts illustrating the ratio of other genomes’ genera that share the same transferred genes. Furthermore, users can inspect the external links connecting to the website page of Pfam, COG and KEGG database, virulence factor and antimicrobial resistance information by clicking ‘Detail’. Like version 1, the HGT relationship plot related to each genome's HGT events and their corresponding phylogenetic trees are concisely provided, along with the summary of HGT-related genomes in graphs and the world map. Lastly, with the Jbrowse program (46), gene clusters adjacent to the selected genes can be seen. The Jbrowse graph will give biological insights to the users about clusters of horizontally transferred genes by checking whether the surrounding genes are related to each other since HGT could often occur in the form of a gene cluster (or multiple genes) (47).

**Figure 5.**
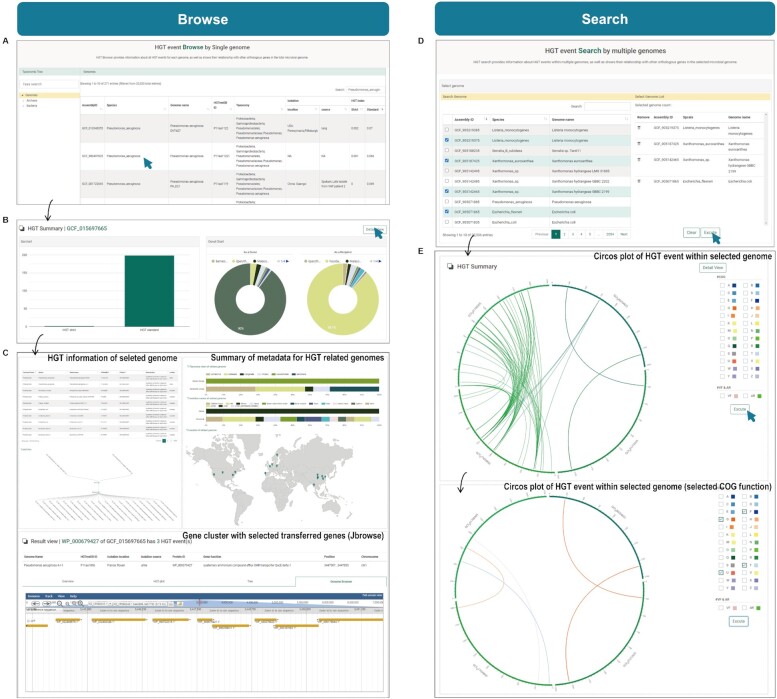
The updated visualization of the HGTree v2.0 interface. (**A**) In the ‘Browse’ section of the website, users can choose a GCF ID to find related HGT event. By clicking the genome, users can check the genome's taxonomy, isolation source, geographical location and the ratio of the HGT-related genes in the genome (the HGT index). (**B**) By clicking ‘Detail’, users can check the ‘HGT Summary’ of the selected genome. The ratio of other genera engaged with the chosen genome by HGT events is shown in the graph. (**C**) Furthermore, the external links connecting to the Pfam, COG and KEGG database are also included for the annotated HGT-related genes, along with the phylogenetic tree concisely showing the movement of the genes. The summary of HGT-related genomes is also shown in a graph and the world map. With the ‘Jbrowse’ program, users can also investigate gene clusters with selected transferred genes. (**D**) The ‘Search’ section provides information on HGT events only in the selected genomes. (**E**) The ‘Circos’ visualization can provide an intuitive understanding of the transferring genes by lines matched with the colors of the donor chromosomes. The lines (HGT events) can be filtered by choosing the COG clusters, virulence factors or antimicrobial resistance genes to only illustrate HGT events that involve genes with such functions.

Unlike the ‘Browse’ section, the ‘Search’ section only shows HGT events in the selected genomes. Within this section, we utilized the program ‘Circos’ (48) to visualize the predicted HGT events between organisms to make it easier for users to comprehend the events and their related genes. As shown in Figure 5, the visualization can provide an intuitive understanding of the transferring genes by pointing out the start location (in the donor's chromosome) and the input location (in the recipient's chromosome) with different colors matched with selected donor genomes. Users can choose the COG clusters, KEGG pathway clusters, virulence factor and antimicrobial resistance to only illustrate those horizontally transferred genes with corresponding functions to figure out what sort of genes could have affected the evolution of microorganisms.

## FUTURE WORKS AND CONCLUSION

In our future research, we intend to concentrate on constructing a database that can additionally accommodate data of other types of microorganisms that can demonstrate HGT, such as viruses, fungi and other eukaryotes. Future works will also involve finding a method to predict HGT-related genes at the strain level since the major drawback of our approach was that we employed 16S rRNA sequences when constructing the species phylogenetic trees. 16S rRNA gives similar (or even identical) sequences for organisms which are more closely related (49); thus, more research into finding an adequate method to specifically clarify species trees at the strain level is still necessary before obtaining more varied information on horizontally transferred genes.

In summary, the HGTree database was updated with a more extensive number of prokaryotic datasets and predicted a much larger number of HGT-related genes than it could do before. Changes in the prediction procedures allowed more reliable detection of putative HGT-related genes. The website interfaces are now more user-friendly with various types of visualization equipment.

## DATA AVAILABILITY

The data are available at http://hgtree2.snu.ac.kr.

## References

[1] Woese C.R. Bacterial evolution. Microbiol. Rev. 1987; 51:221–271. 2439888 10.1128/mr.51.2.221-271.1987 PMC373105

[2] Keeling P.J. , Palmer J.D. Horizontal gene transfer in eukaryotic evolution. Nat. Rev. Genet. 2008; 9:605–618. 18591983 10.1038/nrg2386

[3] Kunin V. , Ouzounis C.A. The balance of driving forces during genome evolution in prokaryotes. Genome Res. 2003; 13:1589–1594. 12840037 10.1101/gr.1092603 PMC403731

[4] Vogan A.A. , Higgs P.G. The advantages and disadvantages of horizontal gene transfer and the emergence of the first species. Biol. Direct. 2011; 6:1. 21199581 10.1186/1745-6150-6-1 PMC3043529

[5] Doolittle W.F Phylogenetic classification and the universal tree. Science. 1999; 284:2124–2128. 10381871 10.1126/science.284.5423.2124

[6] Jeong H. , Sung S., Kwon T., Seo M., Caetano-Anolles K., Choi S.H., Cho S., Nasir A., Kim H. HGTree: database of horizontally transferred genes determined by tree reconciliation. Nucleic Acids Res. 2016; 44:D610–D619. 26578597 10.1093/nar/gkv1245 PMC4702880

[7] Garcia-Vallve S. HGT-DB: a database of putative horizontally transferred genes in prokaryotic complete genomes. Nucleic Acids Res. 2003; 31:187–189. 12519978 10.1093/nar/gkg004 PMC165451

[8] Podell S. , Gaasterland T., Allen E.E. A database of phylogenetically atypical genes in archaeal and bacterial genomes, identified using the DarkHorse algorithm. BMC Bioinf. 2008; 9:419. 10.1186/1471-2105-9-419 PMC2573894 18840280

[9] Ragan M.A. On surrogate methods for detecting lateral gene transfer. FEMS Microbiol. Lett. 2001; 201:187–191. 11470360 10.1111/j.1574-6968.2001.tb10755.x

[10] Sevillya G. , Adato O., Snir S. Detecting horizontal gene transfer: a probabilistic approach. BMC Genomics. 2020; 21:106. 32138652 10.1186/s12864-019-6395-5 PMC7057450

[11] Sánchez-Soto D. , Agüero-Chapin G., Armijos-Jaramillo V., Perez-Castillo Y., Tejera E., Antunes A., Sánchez-Rodríguez A. ShadowCaster: compositional methods under the shadow of phylogenetic models to detect horizontal gene transfers in prokaryotes. Genes. 2020; 11:756. 32645885 10.3390/genes11070756 PMC7397055

[12] Adato O. , Ninyo N., Gophna U., Snir S. Detecting horizontal gene transfer between closely related taxa. PLoS Comput. Biol. 2015; 11:e1004408. 26439115 10.1371/journal.pcbi.1004408 PMC4595140

[13] Li X. , Tong W., Wang L., Rahman S.U., Wei G., Tao S. A novel strategy for detecting recent horizontal gene transfer and its application to Rhizobium strains. Front. Microbiol. 2018; 9:973. 29867876 10.3389/fmicb.2018.00973 PMC5968381

[14] Shikov A.E. , Malovichko Y.V., Nizhnikov A.A., Antonets K.S. Current methods for recombination detection in bacteria. Int. J. Mol. Sci. 2022; 23:6257. 35682936 10.3390/ijms23116257 PMC9181119

[15] O'Leary N.A. , Wright M.W., Brister J.R., Ciufo S., Haddad D., McVeigh R., Rajput B., Robbertse B., Smith-White B., Ako-Adjei D. et al. Reference sequence (RefSeq) database at NCBI: current status, taxonomic expansion, and functional annotation. Nucleic Acids Res. 2016; 44:D733–D745. 26553804 10.1093/nar/gkv1189 PMC4702849

[16] Wheeler D.L. , Barrett T., Benson D.A., Bryant S.H., Canese K., Chetvernin V., Church D.M., Dicuccio M., Edgar R., Federhen S. et al. Database resources of the National Center for Biotechnology Information. Nucleic Acids Res. 2007; 36:D13–D21. 18045790 10.1093/nar/gkm1000 PMC2238880

[17] Chaumeil P.-A. , Mussig A.J., Hugenholtz P., Parks D.H. GTDB-Tk: a toolkit to classify genomes with the Genome Taxonomy Database. 2020; Oxford University Press. 10.1093/bioinformatics/btz848 PMC7703759 31730192

[18] Tabari E. , Su Z. PorthoMCL: parallel orthology prediction using MCL for the realm of massive genome availability. Big Data Analytics. 2017; 2:4. 33312711 10.1186/s41044-016-0019-8 PMC7731588

[19] Lagesen K. , Hallin P., Rødland E.A., Stærfeldt H.-H., Rognes T., Ussery D.W. RNAmmer: consistent and rapid annotation of ribosomal RNA genes. Nucleic Acids Res. 2007; 35:3100–3108. 17452365 10.1093/nar/gkm160 PMC1888812

[20] Potter S.C. , Luciani A., Eddy S.R., Park Y., Lopez R., Finn R.D. HMMER web server: 2018 update. Nucleic Acids Res. 2018; 46:W200–W204. 29905871 10.1093/nar/gky448 PMC6030962

[21] Sievers F. , Higgins D.G. Clustal Omega, accurate alignment of very large numbers of sequences. Multiple sequence alignment methods. 2014; Springer 105–116. 10.1007/978-1-62703-646-7_6 24170397

[22] Price M.N. , Dehal P.S., Arkin A.P. FastTree 2—approximately maximum-likelihood trees for large alignments. PLoS One. 2010; 5:e9490. 20224823 10.1371/journal.pone.0009490 PMC2835736

[23] Bansal M.S. , Kellis M., Kordi M., Kundu S. RANGER-DTL 2.0: rigorous reconstruction of gene-family evolution by duplication, transfer and loss. Bioinformatics. 2018; 34:3214–3216. 29688310 10.1093/bioinformatics/bty314 PMC6137995

[24] Mistry J. , Chuguransky S., Williams L., Qureshi M., Salazar G.A., Sonnhammer E.L.L., Tosatto S.C.E., Paladin L., Raj S., Richardson L.J. et al. Pfam: the protein families database in 2021. Nucleic Acids Res. 2021; 49:D412–D419. 33125078 10.1093/nar/gkaa913 PMC7779014

[25] Buchfink B. , Xie C., Huson D.H. Fast and sensitive protein alignment using DIAMOND. Nat. Methods. 2015; 12:59–60. 25402007 10.1038/nmeth.3176

[26] Camacho C. , Coulouris G., Avagyan V., Ma N., Papadopoulos J., Bealer K., Madden T.L. BLAST+: architecture and applications. BMC Bioinformatics. 2009; 10:421. 20003500 10.1186/1471-2105-10-421 PMC2803857

[27] Chen L. VFDB: a reference database for bacterial virulence factors. Nucleic Acids Res. 2005; 33:D325–D328. 15608208 10.1093/nar/gki008 PMC539962

[28] Alcock B.P. , Raphenya A.R., Lau T.T.Y., Tsang K.K., Bouchard M., Edalatmand A., Huynh W., Nguyen A.V., Cheng A.A., Liu S. et al. CARD 2020: antibiotic resistome surveillance with the comprehensive antibiotic resistance database. Nucleic Acids Res. 2020; 48:D517–D525. 31665441 10.1093/nar/gkz935 PMC7145624

[29] Huerta-Cepas J. , Szklarczyk D., Heller D., Hernández-Plaza A., Forslund S.K., Cook H., Mende D.R., Letunic I., Rattei T., Jensen L.J. et al. eggNOG 5.0: a hierarchical, functionally and phylogenetically annotated orthology resource based on 5090 organisms and 2502 viruses. Nucleic Acids Res. 2019; 47:D309–D314. 30418610 10.1093/nar/gky1085 PMC6324079

[30] Cantalapiedra C.P. , Hernández-Plaza A., Letunic I., Bork P., Huerta-Cepas J. eggNOG-mapper v2: functional annotation, orthology assignments, and domain prediction at the metagenomic scale. Mol. Biol. Evol. 2021; 38:5825–5829. 34597405 10.1093/molbev/msab293 PMC8662613

[31] Galperin M.Y. , Wolf Y.I., Makarova K.S., Vera Alvarez R., Landsman D., Koonin E.V. COG database update: focus on microbial diversity, model organisms, and widespread pathogens. Nucleic Acids Res. 2021; 49:D274–D281. 33167031 10.1093/nar/gkaa1018 PMC7778934

[32] Seemann T. Prokka: rapid prokaryotic genome annotation. Bioinformatics. 2014; 30:2068–2069. 24642063 10.1093/bioinformatics/btu153

[33] Seemann T. barrnap 0.9: rapid ribosomal RNA prediction. 2013; Google Scholar.

[34] Smits S.A. , Ouverney C.C. jsPhyloSVG: a javascript library for visualizing interactive and vector-based phylogenetic trees on the web. PLoS One. 2010; 5:e12267. 20805892 10.1371/journal.pone.0012267 PMC2923619

[35] Bostock M. , Ogievetsky V., Heer J. D³ data-driven documents. IEEE Trans. Visual Comput. Graphics. 2011; 17:2301–2309. 10.1109/TVCG.2011.185 22034350

[36] Kim K.Mo , Sung S., Caetano-Anollés G., Han J.Y., Kim H. An approach of orthology detection from homologous sequences under minimum evolution. Nucleic Acids Res. 2008; 36:e110. 18676448 10.1093/nar/gkn485 PMC2553584

[37] Li Li , Stoeckert C.J., Roos D.S. OrthoMCL: identification of ortholog groups for eukaryotic genomes. Genome Res. 2003; 13:2178–2189. 12952885 10.1101/gr.1224503 PMC403725

[38] Gosselin S. , Fullmer M.S., Feng Y., Gogarten J.P. Improving phylogenies based on average nucleotide identity, incorporating saturation correction and nonparametric bootstrap support. Syst. Biol. 2022; 71:396–409. 34289044 10.1093/sysbio/syab060 PMC8830074

[39] Bansal M.S. , Alm E.J., Kellis M. Efficient algorithms for the reconciliation problem with gene duplication, horizontal transfer and loss. Bioinformatics. 2012; 28:i283–i291. 22689773 10.1093/bioinformatics/bts225 PMC3371857

[40] Kundu S. , Bansal M.S. On the impact of uncertain gene tree rooting on duplication–transfer–loss reconciliation. BMC Bioinformatics. 2018; 19:21–31. 30367593 10.1186/s12859-018-2269-0 PMC6101088

[41] Kloub L. , Gosselin S., Fullmer M., Graf J., Gogarten J.P., Bansal M.S. Systematic detection of large-scale multigene horizontal transfer in prokaryotes. Mol. Biol. Evol. 2021; 38:2639–2659. 33565580 10.1093/molbev/msab043 PMC8136488

[42] Ochman H. , Lawrence J.G., Groisman E.A. Lateral gene transfer and the nature of bacterial innovation. Nature. 2000; 405:299–304. 10830951 10.1038/35012500

[43] Soucy S.M. , Huang J., Gogarten J.P. Horizontal gene transfer: building the web of life. Nat. Rev. Genet. 2015; 16:472–482. 26184597 10.1038/nrg3962

[44] Andam C.P. , Gogarten J.P Biased gene transfer in microbial evolution. Nat. Rev. Microbiol. 2011; 9:543–555. 21666709 10.1038/nrmicro2593

[45] Kanehisa M. KEGG: Kyoto Encyclopedia of Genes and Genomes. Nucleic Acids Res. 2000; 28:27–30. 10592173 10.1093/nar/28.1.27 PMC102409

[46] Buels R. , Yao E., Diesh C.M., Hayes R.D., Munoz-Torres M., Helt G., Goodstein D.M., Elsik C.G., Lewis S.E., Stein L. et al. JBrowse: a dynamic web platform for genome visualization and analysis. Genome Biol. 2016; 17:66. 27072794 10.1186/s13059-016-0924-1 PMC4830012

[47] Dilthey A. , Lercher M.J. Horizontally transferred genes cluster spatially and metabolically. Biol. Direct. 2015; 10:72. 26690249 10.1186/s13062-015-0102-5 PMC4687082

[48] Krzywinski M. , Schein J., Birol I., Connors J., Gascoyne R., Horsman D., Jones S.J., Marra M.A. Circos: an information aesthetic for comparative genomics. Genome Res. 2009; 19:1639–1645. 19541911 10.1101/gr.092759.109 PMC2752132

[49] Ward D.M. , Ferris M.J., Nold S.C., Bateson M.M. A natural view of microbial biodiversity within hot spring cyanobacterial mat communities. Microbiol. Mol. Biol. Rev. 1998; 62:1353–1370. 9841675 10.1128/mmbr.62.4.1353-1370.1998 PMC98949

